# The Kynurenine Pathway As a Novel Link between Allergy and the Gut Microbiome

**DOI:** 10.3389/fimmu.2017.01374

**Published:** 2017-11-06

**Authors:** Aaron P. Van der Leek, Yarden Yanishevsky, Anita L. Kozyrskyj

**Affiliations:** ^1^Department of Pediatrics, University of Alberta, Edmonton, AB, Canada; ^2^Department of Obstetrics and Gynecology, University of Alberta, Edmonton, AB, Canada; ^3^Department of Public Health Sciences, University of Alberta, Edmonton, AB, Canada

**Keywords:** kynurenine, tryptophan, allergy, gut microbiome, indoleamine 2,3 dioxygenase

## Abstract

In the past few decades, the indoleamine 2,3 dioxygenase (IDO) subset of the kynurenine (KYN) pathway of tryptophan (TRP) metabolism has been the subject of much research in the area of immune tolerance. In this review, we aim to incorporate new findings on this pathway in relation to allergy and the gut microbiome, while providing a comprehensive overview of the pathway itself. Stimulated by interferon gamma, IDO acts as a tolerogenic, immunosuppressive enzyme to attenuate allergic responses by the induction of the KYN-IDO pathway, resultant depletion of TRP, and elevation in KYN metabolites. Acting through the aryl hydrocarbon receptor, KYN metabolites cause T-cell anergy and apoptosis, proliferation of Treg and Th17 cells, and deviation of the Th1/Th2 response, although the outcome is highly dependent on the microenvironment. Moreover, new evidence from germ-free mice and human infants shows that gut microbiota and breast milk are key in determining the functioning of the KYN-IDO pathway. As such, we recommend further research on how this pathway may be a critical link between the microbiome and development of allergy.

## Introduction

The indolamine 2,3-dioxegenase (IDO) subset of the kynurenine (KYN) pathway of tryptophan (TRP) degradation has long been acknowledged to contribute substantially to the control of general inflammation (1). As we learn more about TRP metabolism, TRP’s immunomodulatory role in infection, pregnancy, autoimmunity, and neoplasia is increasingly solidified. One area of particular interest is TRP metabolism and its role in allergic disease. Throughout the course of this review, we hope to provide a careful evaluation of TRP metabolism and of its link between allergy and the gut microbiome. One of the key components of this argument is the hygiene hypothesis, which at its simplest postulates that less microbial exposure during early-life drives the immune system in a T-helper (Th) type 2 direction (promoting an allergic response) away from a balanced Th1/Th2 state (2). Early microbial exposure and resultant stimulation of the KYN-IDO pathway by Toll-like receptors (TLR) may work in conjunction with the hygiene hypothesis to determine the outcome of allergy, autoimmunity, and other disease (3). Current evidence is promising to suggest that induction of the KYN-IDO pathway and resultant depletion of serum TRP and increase of TRP metabolites controls the allergic state (4). This evidence, along with knowledge on the mechanism for IDO activation and for the induction of tolerance *via* IDO, makes it very likely that the KYN-IDO pathway is a major player in the control and development of allergic disease.

## Tryptophan

Tryptophan is one of nine essential amino acids supplemented by our diet, with an estimated dietary requirement of 5 mg/kg/day. It is a non-polar, α-amino acid used for the biosynthesis of proteins and a precursor to a number of biologically important compounds—serotonin, melatonin, tryptamine, niacin, and auxins. Foods that contain high amounts of TRP include: eggs, cereal, soybeans, kiwis, plums, walnuts, milk, meat, eggplants, and tomatoes (5). Inadequate dietary intake of TRP has resulted in a number of diseases, with pellagra, characterized by dermatitis, diarrhea, and cognitive malfunction, the best studied. Essential to understanding the immunomodulatory effects of TRP, Box 1 describes basic elements of its fate in the body and the KYN pathway.

Box 1 **Tryptophan (TRP) pharmacokinetics.**

**Absorption**

**Figure 1:**
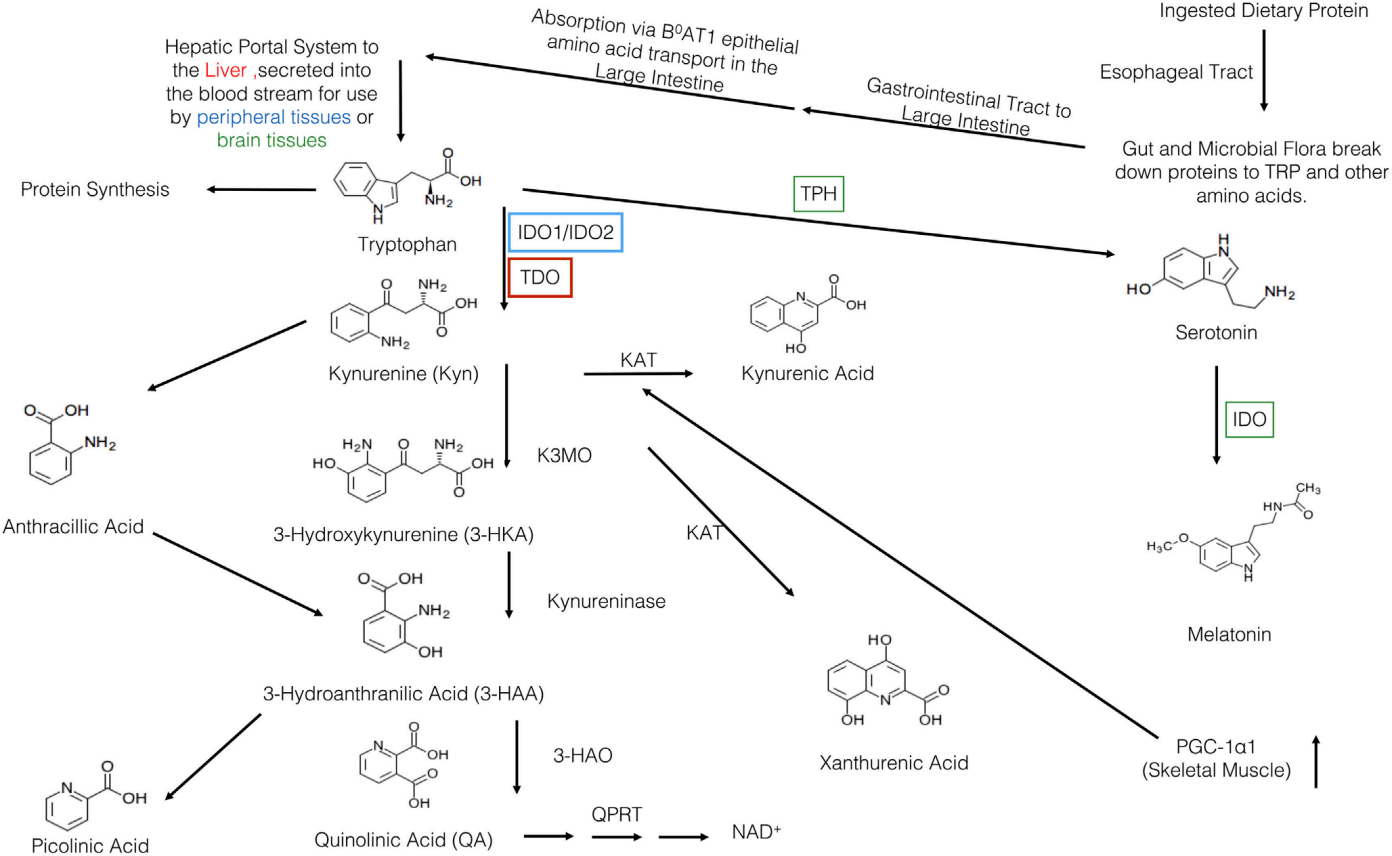
Overview of the kynurenine pathway (KP). Protein is first ingested and travels to the gut where is broken down into tryptophan (TRP) and other amino acids. Then, it travels to the large intestine where it is absorbed by the B^0^AT1 epithelial amino acid transport system. TRP, then, travels through the hepatic portal system to the liver where it is used or secreted into the blood stream for use in the peripheral or brain tissues. TRP has three major metabolic pathways: incorporation into proteins, production of serotonin, or the breakdown *via* the KP. The various KYN metabolites are either neurotoxic, neuroprotective, and/or immunomodulatory. Within the KP, there are two major breakdown areas: the hepatic route where TDO is the rate-limiting enzyme and the peripheral route where IDO-1/IDO-2 are the rate-limiting enzymes. Within the peripheral route, only dendritic cells, macrophages, microglia, eosinophils, fibroblasts, and endothelial cells have been show to contain IDO. In recent years, there have been a number of links between IDO, KYN, and various KP metabolites to various allergic diseases. Recently, Ruas et al., have also shown a connection between activation of skeletal muscle PGC-1α1 and modulation of the KP resulting in resiliency to stress-induced depression. Although this pathway may be a critical link between depression, allergic disease, and exercise, it has yet to be elucidated.

Subsequent to ingestion, TRP is first received by the gut and resident microbiota (Figure 1). There, both the microbiota and the enterochromaffin cells of the gut have the first physiologically relevant interaction with ingested TRP. Past the gut and through to the large intestine, absorption of ingested TRP occurs primarily on the apical membrane of the intestinal enterocytes and is mediated *via* the B^0^AT1 epithelial amino acid transport system (6). This system is responsible for the absorption of most other neutral amino acids, all of which have a higher affinity for this transport system then TRP. This competition for absorption can cause malabsorption of TRP (6). Subsequent to absorption *via* enterocyte transporters, TRP travels the hepatic portal system where it is utilized by the liver for protein synthesis or NAD^+^ production *via* the kynurenine (KYN) pathway (7). Unused TRP is then secreted into the blood stream and is available for use by peripheral tissues. Common cell types utilizing TRP include vascular endothelial cells, fibroblasts, and antigen-presenting cells (APCs), more specifically, dendritic cells, monocytes, and macrophages (8, 9). Besides uptake by peripheral cells, TRP can also be transported across the blood–brain barrier and this transport is critical to the regulation of serotonin synthesis, a process based on competitive transport shared by several large neutral amino acids (10). Hence, higher plasma TRP levels result in increased TRP uptake and serotonin synthesis. Uptake of TRP by peripheral cells such as tissue macrophages may affect the rate of absorption in the liver, but this link is not yet fully understood (11).

**Metabolism**

Once ingested, TRP faces a plethora of metabolic fates (Figure 1). In the gut, TRP is exposed to billions of microorganisms that utilize TRP for the synthesis of serotonin and other metabolically active compounds (90% of the serotonin in the body is produced by the gut microbes and cells lining the gut) (12). In addition to microbes in the gut, serotonin is produced by enterochromaffin cells lining the gut. These cell types carry the rate-limiting enzyme TRP hydroxylase-1, which converts TRP into 5-hydroxytryptophan, a metabolite which is subsequently decarboxylized to 5-hydroxytryptamine, 5-HT, or serotonin. In terms of the physiological relevance of its production in the gut, serotonin is a critical response to various mechanical, pathological, and chemical stimuli in the lumen. The serotonin produced in the gut is needed for secretory and peristaltic reflexes, and it also activates vagal afferents through serotonin_3_ receptors that signal to the brain to generate feelings of nausea (12). Once absorbed by the intestines and transported to the liver, l-TRP faces a number of other metabolic pathways.

The liver is the second stop for TRP eukaryotic cellular utilization after the gut, but it arguably has the most significant influence on TRP availability and utilization throughout the rest of the body than any other tissues. Within the liver, TRP faces two primary fates: protein synthesis and degradation *via* the KYN pathway. The KYN pathway is present both in the periphery and liver, catabolizing TRP to yield the essential cellular cofactor, nicotinamide adenine dinucleotide (NAD^+^); this happens in the event of low niacin in the diet and also yields a multitude of physiologically active/relevant catabolites throughout the metabolic process (13). The general pathway sequence is as follows in Figure 1 and below: the indole ring of l-TRP is broken by TRP 2,3-dioxygenase (TDO) to produce *N*-formyl KYN. *N*-formyl KYN is further broken down into l-KYN (l-KYN) by formamidase. l-KYN then faces four different fates because it is used as a substrate by four different enzymes: kynurenase (forming anthracillic acid), KYN 3-hydroxylase (forming 3-hydroxykynurenine), and KYN aminotransferases KAT 1 and KAT II (forming kynurenic acid). Anthracillic acid and 3-hydroxykynurenic acid progress to 3-hydroanthranilic acid (3-HAA), which can be transformed into picolinic acid or quinolinic acid. Quinolinic acid is further metabolized into nicotinic acid dinucleotide (NAD) by quinolinate phosphoribosyl transferase. NAD is then further metabolized to reach NAD^+^ which is an essential coenzyme found in all cells primarily used for electron transfer (14).

### TRP Breakdown by KYN Enzymes

The full library of KYN enzymes is only known to be fully expressed in hepatocytes, tumor cells, vascular endothelial cells, fibroblasts, and antigen-presenting cells (APCs), specifically, dendritic cells, monocytes, and macrophages (8, 9). It is critical to note that hepatocytes differ from other cells by the type of the rate-limiting enzyme of the TRP pathway; TRP 2,3-dioxygenase (TDO) is found in the liver, as opposed to indolamine 2,3-dioxegenase 1 or 2 (IDO-1 or IDO-2) which are found elsewhere. Functionally IDO and TDO are similar but are regulated in a very different fashion, resulting in different physiologic roles. Since TDO has a different mechanism for activation and is activated by TRP itself, TDO is almost always continuously activated and expressed; because of this, it is subsequently responsible for 90% of TRP degradation (15). When there is an accumulation of one of the intermediate products of the pathway, this causes inactivation of TDO and a succeeding increase in serum TRP and in TRP availability to the rest of the body.

Comparatively, the rate-limiting enzymes present in cells other than hepatocytes are either IDO-1 or IDO-2, which are similar in structure but vary by signaling pathways and expression patterns (16). IDO-1 is the predominant of the two enzymes and is found in a large number of cell types including, but not limited to, astrocytes, neurons, microglia, dendritic cells, monocytes, and macrophage, whereas IDO-2 has only been found in a smaller subset of cells, primarily dendritic and stem cells, and some cancer lines (16–21). IDO-1/IDO-2 is the enzyme responsible for catalyzing the rate-limiting step in the peripheral tissues and are dependent upon the active form of superoxide $\left({\text{O}}_{2}^{-}\right)$. Various co-inducers are also required for their activation and expression. They include cytokines (IFN, TNFα, TGF-β), lipo-polysaccharides like amyloid peptides, some human immunodeficiency proteins (HIV), and various TLR ligands. The most notable and potent inducer of IDO is the pro-inflammatory cytokine interferon gamma (IFN- γ) (13, 22–24). IFN-γ influences both the activation and the expression of IDO-1. Although not much is known about IDO-2, it was initially hypothesized to be preferentially inhibited by d-1-methyltryptophan (19, 25), an adjuvant chemotherapeutic agent, suggesting a roll of IDO-2 in tumorigenesis (13, 22–24). Despite this, recent evidence suggests that d-1-methyltryptophan does not actually inhibit IDO-2 activity but affects the pathway by acting as a TRP mimetic. As such, d-1-methyltryptophan exerts its effect on essential amino acid metabolic pathways *via* mTOR (mechanistic target of rapamycin) and general control non-depressible 2 (GCN2) (general control nonderepressible 2), the end results of these cellular pathways controlling inflammatory responses and immune tolerance (26). Further studies have also shown that d-1-methyltryptophan should not be referred to as an inhibitor (26–29). As such, additional work has to be done to solidify IDO-2’s differential role from the IDO-1 isoform.

Regarding TRP utilization by each pathway, 95% of ingested TRP is broken down *via* the KYN pathway, 1–2% is used for protein synthesis and, 1–2% for serotonin synthesis (30). Succeeding the TDO-KYN and IDO-KYN pathways, any unused TRP then crosses the blood–brain barrier by competitive large neutral amino acid transport (10). In parallel with the gut, the rate-limiting enzyme is TRP hydroxylase-1 converts TRP into 5-hydroxytryptophan, which is then decarboxylized to 5-hydroxytryptamine (or serotonin). Subsequent to serotonin production, the pineal gland can further process serotonin into melatonin (10). Derangements in any of these TRP degradation steps can lead to altered metabolism of serotonin, melatonin, and KYN. Through the study of malfunctioning pathways, it is becoming increasingly apparent that TRP catabolism is a key player in the pathogenesis of gastrointestinal, cardiovascular, respiratory, allergic, neurodegenerative, and psychiatric disorders (30).

Contrasting IDO and TDO’s mechanisms of activation, immune-related activation versus feedback control provides an obvious link between IDO and the immune system, which has become a major research focus. While evidence points to immune tolerance-related properties of the KYN-IDO pathway, there is certainly competing evidence (31). The comprehensive review by Yeung et al. chronicles the ever-growing list of immunomodulatory activity of this pathway and its importance to health and disease (32). In terms of this review, we will be focusing on the KYN pathway in relation to allergic disease. Two competing theories and various interlinking pathways will be discussed. Ultimately, we will attempt to show that the KYN pathway may connect the development of allergic disease to the gut microbiome in infancy.

## TRP Metabolism and Its Immunomodulatory Functions

### TRP Degradation versus Pathway Metabolites

The general function of the host immune system is to correctly distinguish between self and non-self and to initiate a protective cascade of immune responses in the presence of a harmful, non-self-antigen. For obvious reasons, this balance between the initiation and suppression of the immune response is dependent upon a high number of regulator mechanisms, one of which is the KYN pathway. Concerning this delicate balance of initiation and suppression, a growing body of research from the turn of the century has identified in particular, the IDO subset of the KYN pathway as having an active role in maintaining equilibrium between the two responses.

Although the primary link and majority of research on the immune function of the IDO is on the rate-limiting enzyme IDO-1 (herein referred to IDO due to its central role), it is critical to compare it to TDO. Since an obvious immunological link comes from the mechanism of activation of these enzymes, this is where we will start. As mentioned, only hepatocytes possess the rate-limiting enzyme TDO and due to its mechanism of activation (by TRP itself) and localization (in the liver, the first destination for TRP after absorption), it is consequently responsible for the majority of TRP degradation in the body (~90%) (33). Figure 1 shows the general schematic of the KYN pathway. In comparison to the IDO-KYN pathway that is localized primarily to the lymph nodes and inflammatory tissue, the TDO-KYN pathway has no major known immune functions/links. However, it controls TRP availability in the rest of the body, and this can cause downstream effects by limiting TRP availability to IDO. This TRP homeostatic property of the KYN pathway is reinforced by findings showing that the concentration of TRP itself influences TDO activity by stabilizing the enzyme complex (34). Studies on the immunological function of TDO have been limited and primarily to *in vitro* studies. While they show a limited antimicrobial and immunological function of TDO *via* TRP depletion, it still remains to be seen whether these studies are applicable to what happens *in vivo* (35). More recent (2016), *in vivo* studies in knock-out mice have confirmed a role for TDO in mediating control of basal KYN pathway metabolites and NAD production, and of IDO in situations where the immune system is activated (36). As an interesting aside, evidence suggests a compensatory mechanism between IDO and TDO; when the one is knocked out in mice, the other one seems to compensate for the other’s absence and functions (36, 37).

Functional IDO is observed in human CD123^+^CCR6^+^ plasmacytoid dendritic cells, regulatory B-cells, activated monocytes, and macrophages, and also in NK cells, eosinophils, and neutrophils (15). The IDO enzyme causes cellular depletion of TRP and accumulation of biologically active KYN pathway metabolites (31). The majority of the regulatory actions of the IDO pathway act primarily on T-cells, NK cells, dendritic cells, and macrophages. In T-cells, IDO promotes regulatory T-cell differentiation, induction of apoptosis in various subsets and T-cell receptor (TCR) activation. IDO’s actions on NK cells cause downregulation of activating receptors and cell death. IDO has also been shown to control dendritic cells maturation and migration (38).

The first discovery of immunomodulatory effects of IDO was unknowingly made in 1984 when Pfefferkorn observed that IFN-γ induced TRP degradation and blocked growth of *Toxoplasma gondii* in human fibroblasts*;* what he actually observed was IFN-γ activation of IDO and the induction of the KYN pathway, TRP breakdown and accumulation of KYN metabolites (39, 40). These observed immuno-regulatory effects were initially attributed to local cellular depletion of TRP. Appropriately called the *TRP Depletion Theory*, this first TRP-immunoregulatory theory posited that activation of IDO results in a local cellular depletion of TRP that serves two major functions (1): TRP starvation of microbes, causing death and (2) promoting Th2 cell polarization. Generally, a Th2 polarization mediates both the activation and maintenance of humoral (antibody-mediated) immunity against extracellular bacteria, allergens, toxins, and parasites. This is in contrast to a Th1 polarization that mediates cellular immunity and plays an important role for host defense systems in resistance against intracellular microbial agents and viruses. Additionally, Th1 polarization is also associated with autoimmunity and inflammation.

Newer studies have shown that TRP depletion as the root cause for immunomodulatory outcomes has been difficult to prove (41). In spite of this, there appears to be some merit to this theory. Recent evidence suggests that the immunomodulatory properties of IDO are largely due to the accumulation of KYN metabolites in conjunction with TRP depletion (42). Although not specifically attributed to the build-up of KYN metabolites, a study by Mellor et al. (43) helped catalyze the notion that immunomodulation of the IDO pathway in peripheral cells is more than TRP depletion. Mellor et al.’s study showed in pregnant mice that the murine allogenic conceptus was protected from T-cell-mediated rejection when IDO was expressed at the fetal/maternal interface. Stemming from this initial study, a large body of research on IDO’s T-cell suppression immunomodulatory effects has pointed to the general trends that are summarized in Ref. (32).

Along with the Mellor et al.’s study on allogenic fetuses, early *in vitro* studies pointed to a regulatory subset of IDO-expressing DCs and macrophages that have the ability to induce cell cycle arrest in T-cells dependent on IDO metabolism (44–46). These initial studies attributed inhibition of T-cell responses to the prevention of TCR activation. More recent evidence indicates that TRP starvation *via* IDO does not solely act *via* TCR inactivation but in conjunction with induction of FAS-mediated cell cycle arrest in the mid G1 phase of T-cell apoptosis, clonal anergy, and inhibition of antigen-specific T-cell responses (1, 30, 47, 48). Consistent with the TRP depletion theory, many early studies reported immunosuppression following TRP deprivation and restoration of effector T-cell activity with the addition of excess TRP. In addition, reduced levels of TRP in T-cells have been shown to be correlated with elevated uncharged tRNA levels, activation of the GCN2 kinase pathway, and inhibition of mTOR (mammalian target of rapamycin) and PKC (protein kinase C) signaling pathways, shown to promote T-cell autophagy and anergy (49). All of these effects of TRP depletion result in a stress–response program that leads to cell cycle arrest, differentiation, adaptation, or apoptosis of T-cells (50–52). While they tentatively explain some of the immunosuppressive effects mediated by IDO-TRP deprivation (1, 53), the accumulation of the KYN pathway metabolites has been greatly ignored in these studies.

### Activity of KYN Pathway Metabolites

In the past, KYN catabolites were viewed as inert precursors for the formation of NAD^+^, but newer findings have identified a number of physiological effects for these metabolites. The addition of exogenous KYN metabolites (KYN, 3-HAA, QA, 3-HK, and picolinic acid) to various immune cell subtypes showed that KYN metabolites can selectively inhibit active T-cells, B-cells, and NK cells at more physiologically relevant TRP levels (specifically in the case of T-cell suppression) than the TRP depletion theory would suggest (54–57). The IDO enzyme in TGFβ-treated murine plasmacytoid dendritic cells is able to form an intracellular scaffold that binds both SHP-1 and SHP-2 protein tyrosine phosphates to create a cellular signal for long-term immune tolerance by converting CD4^+^ T-cells into immunosuppressive FoxP3^+^ Tregs cells (53).

Although several *in vitro* studies convincingly show the immunopsuppressive effects of IDO-expressing cells when TRP concentrations are below <0.5–1 μM, many cell types remain viable and functional in low TRP environments. Part of this inconsistency is attributed to differential expression of tryptophanyl-tRNA synthetase (TTS) which catalyzes TRP binding with tRNA for incorporation into proteins; cells with high expression of TTS have greater protection from external stress by sequestering sufficient TRP for cellular protein synthesis (58). Further, it is possible that although TRP levels in peripheral tissues are greatly reduced, IDO has sufficient available TRP to remain functional due to higher circulating plasma TRP levels *in vivo* (ranging from 40 to 100 µM) that readily diffuse into the tissues, as per the TTS sequestering system.

While a few general trends have emerged, the physiological effects of KYN metabolites appear to be more complex and nuanced than simple initiation or suppression. Most of this research is based on the actions of KYN metabolites as endogenous ligands of the aryl hydrocarbon receptor (AhR). Initial research concerning AhR was on KYN itself. In these *in vitro* studies, KYN promoted differentiation of naive CD4^+^ T-cells into immunosuppressive FoxP3^+^ Tregs and not pro-inflammatory Th17 cells (59); KYN also promoted IDO expression in dendritic cells (60, 61). Other metabolites of the KYN pathway have demonstrated activity as AhR ligands, but differently from KYN. Kynurenic acid (KYNA) binds to AhR to produce the pro-inflammatory cytokine, IL-6 (62). Meanwhile the 3-HAA metabolite has been observed to cause immune suppression by inducing apoptosis in T-cells through glutathione depletion; 3-HAA administration was effective in reducing rejection of allogenic bone marrow transplantation—signaling a tolerogenic effect—and inducing apoptosis in Th1 but not Th2 cells (52). Many of the affected cells also engage in a cross talk that helps establish and amplify immunosuppressive signals. Examples of this include IDO-expressing plasmacytoid dendritic cells converting CD4^+^ T-cells to FoxP3^+^ Tregs and in turn, Tregs inducing IDO expression in plasmacytoid dendritic cells and neutrophils (54, 63, 64).

One of the most interesting aspects of the KYN-IDO pathway is that induction of IDO almost invariably leads to preferential apoptosis of Th1 cells. Consensus is that IDO is critical for regulating the immune system by reducing Th1 cell proliferation and inducing differentiation of Treg cells. The end result is the maintenance of immunological tolerance while also limiting tissue damage. Although this explanation appears to be complete, the outcome of inducing the IDO pathway on Th2 cells is substantially more complex with both inhibitory and stimulatory actions reported (31). Initial evidence for Th2 cell regulation indicated stimulation, with IDO-expressing human eosinophils preferentially inhibiting Th1 cells but promoting Th2 cells (65). A similar study of murine spleen cells also showed that IDO expression and the addition of exogenous TRP resulted in preferential decrease of Th1 cytokine production and resultant increase in Th2 cytokine production (66).

Compared to observations of Th2 cell promotion and Th1 inhibition, *in vivo* studies on allergy have shown contradictory effects for IDO. In a study on ovalbumin-induced asthma, mice that were IDO deficient showed significantly weaker Th2 responses in comparison to WT control mice when challenged with inhaled antigen (4). Serum levels of antigen-specific IgE were also lower when compared to WT mice; in this case, IDO deficiency protected against allergic disease (4). In another murine model of asthma using ovalbumin sensitization, induction of IDO expression inhibited Th2-induced asthma (67). Further studies on Th1/Th2 preferential apoptosis mediated by IDO have shown that Th2 cells are sensitive to TRP metabolic apoptosis and not Th1 (52). Although these contradicting Th2 mechanisms have yet to be fully elucidated, Xu et al. have proposed an explanation that remedies some of the inconsistencies (31). Their thesis is that during the course of a Th1-mediated immune response, apoptosis in Th1 cells is preferentially induced; this contrasts a Th2-mediated immune response, wherein Th2 cells are targeted by the KYN-IDO pathway. As seen in the majority of earlier studies, Th1 cells are killed and Th2 cells proliferate during an immune response that is both Th1 and Th2 mediated. In addition to the effects of TRP on Th1, Th2, and Th17 cells, recent evidence has also shown that TRP catabolites engage the AhR and influence innate lymphocytes (68). Currently, this research only provides a link to ILC3 (group 3 innate lymphocytes) and not ILC2 (group 2 innate lymphocytes which are implicated in allergic disease). Despite this, there may be an indirect link to allergy as ILC3s affects the gut microbial population. Further research addressing these cell types needs to be done to elucidate what may be another mechanism between TRP and its effects on allergy (69–73).

Returning to the topic of T cell balances, Xu et al.’s explanation stems from the 2 initial theories by MacKenzie et al. and Mellor and Munn which offer a more comprehensive explanation for these somewhat seemingly contradictory effects (37, 74). MacKenzie et al.’s thesis centered on a negative feedback regulatory loop for Th1 cell responses. It is well known that as antigens and pathogens are presented by myeloid dendritic cells for T-cell activation, this causes skewing of Th0 cells to Th1 and produces a strong upregulation of IFN-γ release from T cells and NK cells (75, 76). Various other processes have also been shown to produce IFN-γ through response to antigens and pathogens through various TLR receptors and other protein receptors (37, 64, 77–79). In conjunction with existing theories, increased IFN-γ can have several major consequences, the most obvious of which is the creation of a Th1 dominant microenviroment and inhibition of Th2 differentiation. As IFN-γ induces dendritic cells to express functional IDO, there is a reduction in TRP, increase in KYN metabolites and subsequent T-cell inhibition. The majority of findings show preferential induction of apoptosis in Th1 cells and not Th2 due to increased susceptibility of Th1 cells to KYN metabolites (59, 64). Therefore, IDO-mediated inhibition of Th1 cells and selected survival of Th2 cells may act through IFN-γ stimulation as a regulatory loop to limit overactive Th1 cells responses.

The theory by Mellor and Munn also recognizes that KYN promotes differentiation of naive CD4^+^ T-cells (*in vitro*) into immunosuppressive FoxP3^+^ Tregs, as opposed to pro-inflammatory Th17 cells, and that IDO activates Treg cells *via* GCN2 pathways (49). Since these reactions are not as fast or direct as Th1/Th2 differentiation from Th0 cells, IDO-induced Treg proliferation suppresses Th1 and Th2 immune cells, and inhibits an overactive immune response. In sum, it appears that IDO pathways have a tolerogenic role, whereby loss of key functional enzymes in the pathway results in an aberrant response to immune stimuli. The inhibition of a T-cell response or selective T-cell proliferation is highly dependent upon the immediate microenvironment; IDO pathway enzymes and metabolites act selectively to maintain tolerance whether it is a Th1-, Th2-/Th1-, or Th2-mediated immune response.

### The KYN-IDO Link to Allergy

Recognized initially for its tolerogenic and immunoregulatory roles, there is growing evidence that the KYN-IDO pathway plays a major role in development of atopy and allergy. Before detailing the specific actions of IDO-KYN on allergy, it is important to understand some key characteristics of allergic disease, which in essence, is an abnormal reaction of the immune system to an ordinarily harmless substance called an allergen. The first step in the development of allergy is the presentation of an allergen to a T cell by APCs. Together with the APCs, these newly activated Th2 cells secrete various mediators such as IL-4 (positive feedback loop for Th2 cell differentiation), Il-5 (eosinophil activation), and IL-13 (similar in function to IL-4) cytokines. In this Th2-dominated environment, activated Th2 cells communicate with B cells, leading to allergen-specific Immunoglobulin E (sIgE) production. The majority of secreted sIgE (specific IgE) will bind to its high-affinity IgE receptors (FcεRI) on mast cells (tissue-residing) and basophils (in blood). Subsequent exposure to the same and or cross-reactive allergen will lead to cross linking of the FcεRI-bound IgE, initiating cell activation and ultimately the release of various mediators, such as histamine. These mediators cause the hallmark symptoms and signs of an allergic reaction involving various systems such as the respiratory, skin, gastrointestinal, and cardio vascular systems (59, 64). Although FcεRI are usually found on the surface of mast cells and basophils, they are expressed on other immune-type cells like APCs of atopic individuals. Subsequent exposures to the antigen result in the same immediate-type allergic reaction (3).

The hygiene hypothesis argues that insufficient exposure to pathogens in early development leads to insufficient stimulation of Th1 cells and diminished capability to counterbalance an expansion of Th2 cells, resulting in a predisposition to allergy. Since the IDO-KYN pathway can directly affect the Th1/Th2 balance *via* selective apoptosis and Treg stimulation, the IDO pathway is likely critical to the allergic disease process. Further, microbial stimulation of the IDO pathway by TLR may also help determine the outcome of allergic inflammation (49). In atopic individuals, activation of monocytes *via* the FcεRI receptor induces IDO activity resulting in increased TRP metabolism (80). This induction of the IDO pathway and subsequent immune system events help to limit allergic responses, such that individuals remain unresponsive after being exposed to an allergen.

The immunosuppressive role of IDO in response to an allergen was initially realized through a study by Bubnoff Von et al., which employed a technique called suppression subtractive hybridization to identify genes upregulated by the engagement of FcεRI. Sequences coding for KYN 3-monoxygenase and IDO were differentially upregulated in stimulated monocytes when compared to unstimulated monocytes (80). When stimulated through FcεRI, subsequently it was shown that relative amounts of these enzymes were increased to a greater extent in the monocytes of asymptomatic atopics versus symptomatic atopics. The ability to suppress T-cells in atopics was later confirmed to be reliant upon IDO expression and degradation of TRP (81). Using the KYN/TRP ratio in plasma as a marker for IDO activity (82), Bubnoff von et al. found this ratio to be reduced, alongside lower TRP and higher KYN levels, in adults sensitized to aeroallergens who were asymptomatic versus those who exhibited symptoms. Further, a recent study from Buyuktiryaki et al. reported lower serum KYN/TRP ratios in children with food allergy that persisted versus healthy children or in food allergic children who had developed tolerance (83). IDO mRNA expression did not differ between tolerant and persistent food allergic children but IFN-γ and IL-10 synthesis were significantly higher in children tolerant to milk.

Human observational studies suggest that IDO activity can induce tolerance to food or aeroallergens after allergen sensitization occurs. Some of the strongest human evidence for the IDO pathway is in relation to clinical trials of allergen-specific immunotherapy (SIT), which involves administering increasing doses of the substance to which a person is allergic in order to develop tolerance. Initial results from SIT trials documented higher TRP degradation during therapy (80). IDO has subsequently been proven to be partially responsible for tolerance induction during SIT, with KYN metabolites mediating this effect as opposed to TRP depletion (80, 82, 84). In Taher et al.’s Th2-dependent allergic airway model, the suppression of eosinophils and reduction of inflammation to achieve tolerance was dependent upon 3-KYN, KYN, and XA (85).

In summary, most agree that IDO acts as a tolerogenic, immunosuppressive enzyme to reduce allergic inflammation, with the induction of the IDO-KYN pathway, resultant depletion of TRP, and elevation in KYN metabolites. Some studies report pro-inflammatory activity for IDO (86), but they are primarily in IDO knock-out mice in which developmental defects such as impaired Th2 cell function make it challenging to interpret findings (85, 87). Current consensus is that induction of the IDO-KYN pathway causes T-cell anergy (4), T-cell apoptosis (4), proliferation of T-regs and Th17 cells (49), and deviation of the Th1/Th2 response (49). More studies are required to solidify how each of the KYN metabolites acts to create tolerance to various allergens.

### TRP, Serotonin, and Allergy

Allergic disease is often comorbid with depression. Greater induction of the KYN pathway lowers available TRP levels to cross the blood–brain barrier *via* competitive transport (88) and the production of serotonin, a key characteristic of depression. More concretely, allergen exposure in an atopic individual can activate the IDO-KYN pathway to promote tolerance; this enhanced catabolism of TRP reduces serotonin production. Additionally, all immune cells have the capacity to interact with serotonin according to the differential expression of serotonergic components. For example, serotonin directly regulates cell proliferation and cytokine production at the transcriptional level in leukocytes (31).

The KYN pathway and/or accumulation of KYN metabolites have also been shown to contribute to depressive-like behaviors, which can be negated *via* exercise (42). Increasingly, we are appreciating exercise and skeletal muscle breakdown of KYN as a useful pathway to deplete TRP levels and alleviate depression. Fuertig et al.’s murine model of chronic social stress showed that stress incited fear behavior and elevated KYN pathway activity in the blood and brain, while inhibition of IDO reversed both effects (89). Following exercise-induced activation of the skeletal muscle PGC-1α1 pathway in mice, Agudelo et al. found greater expression of KYN aminotransferases and enhanced conversion of KYN into KYNA; as KYNA is unable to cross the blood–brain barrier, a reduction in depressive behavior was observed (90, 91).

It is clear that the KYN pathway has a major influence on the availability of TRP for serotonin production. However, experimental evidence that this pathway can induce fear behavior that is reversible with IDO, and human observations of higher IDO activity in allergy (82, 83) are not consistent with enhanced depression seen in the presence of allergy. There is likely a more complex dialog between immune cells and the brain involving TRP and serotonin levels. Another possibility is that IDO-induction of tolerance in an atopic individual might lead to higher risk for depression. Clearly, the tentative link between allergy and depression has yet to be fully elucidated. In the meantime, the influence of the germ-free state in mice on plasma TRP and serotonin concentrations points to the microbiome as another pivotal link in the mystery of TRP metabolism, its effects on allergy and depression, and takes us to the final section of our review (92).

## KYN Pathway Cross Talk Between the Gut Microbiome and Immune System

Residing in the human gut is our *microbioata*, a large and diverse collection of microorganisms which play a crucial role in regulating host and intestinal health (93). Bacterial cells within the whole human microbiome are more plentiful than human cells, especially in the gut, and their total gene count outnumbers that of the host by more than 100 times (94). The expression of this plethora of genes results in numerous enzymatic reactions with a myriad of physiological outcomes that would otherwise be unavailable to the host. It is for this reason that many now consider the microbiome to be an “organ within an organ” or the “second brain.” Not only is the gut microbiome essential for the digestion, absorption, and energy storage of food substrates, it also supports other immune and neurologic system functions (95).

In earlier studies employing a broad metabolomics approach, the composition of gut microbiota was noted to have a profound influence on circulating metabolites; plasma levels of KYN metabolites were affected to a greater extent than levels of other metabolites (96). In this study and ones that followed in germ-free rodents, plasma TRP levels were elevated in the pre-microbial colonization state, alongside reductions in serotonin and KYN levels (96–99). Serotonin, TRP, and KYN levels were normalized following microbial colonization of mice immediately post-weaning; normalization did not occur in rats. Similarly, Desbonnet et al.’s study of antibiotic-induced microbial depletion in mice reported higher circulating TRP levels and reduced peripheral KYN metabolism (92). Finally, changes to circulating KYN/TRP ratios have been observed following experimental induction of gastrointestinal inflammation *via* introduction of a parasite and likely altered gut microbiota (100).

The lowering of serotonin and KYN metabolite levels in the absence of gut microbiota and their restoration following the re-introduction of gut microbes indicates a key role for gut microbiota in the KYN pathway. More recent murine and human colonocyte studies provide further evidence for the regulation of gastrointestinal synthesis of serotonin by spore-forming microbiota, specifically by their metabolites, the short-chain fatty acids, deoxycholate, alpha-tocopheral, and others (101, 102). Of note, host TRP is required for this biosynthesis. How are these events initiated? We have already discussed the important role of AhR in modulating the immune system and the expression of IDO/TDO (103, 104). In the absence of AhR, endogenous KYNA levels have been documented to rise (105). TLRs are critical to the gut microbial community. In fact, activation of TLRs by microbial components has been identified as a key factor in initiating KYN metabolism. Several studies have linked microbial- induced KYN pathway changes to the expression of colonocyte TLRs in germ-free mice (99, 106, 107), namely, reduced TLR stimulation in germ-free mice has resulted in reduced TRP metabolism.

### Maternal Microbiota, Postnatal Immune Development, and the KYN Pathway

Immediately after birth, our gut microbiota is dominated by the lactic-acid bacteria: bifidobacteria, lactobacilli, and enterococci (108). Postnatal maturation of this microbial community is shaped by breastfeeding and seeded by the maternal vaginal and intestinal microbiomes, which potentially commences *in utero* (109). Interference with this normal biological process alters the gut microbial composition of infants at critical periods of development, with long-lasting sequela on health, including overweight and allergic disease (110, 111). Atypical early gut microbial development alters the ability of the infant’s developing immune system to distinguish between harmful and harmless antigens and slows postnatal maturation of the barrier functions of cells lining the gut (112). As noted above, microbial deficiency and under-stimulation of the immune system in germ-free mice has led to deviant KYN metabolism.

As we have summarized, gut microbial dysbiosis can influence circulating concentrations of TRP and serotonin, making the development of the TRP and KYN pathways, and serotogenic system particularly susceptible to postnatal gut microbial development. Altering gut microbiota through the administration of probiotics in human supplementation trials has also influenced circulating TRP levels. Relevant to the lactic-acid bacterial dominance of the newborn gut, Strasser at al.’s trial documented that when athletes were given a probiotic mixture of bifidobacteria, lactobacilli, and enterococci, drops in TRP levels, seen in control subjects after exercise, were prevented and the incidence of respiratory tract infections was reduced (113). The latter observation also provides added evidence for a role for TRP or the KYN-IDO pathway in TLR recognition of microbes and early-life infection.

Both in human and experimental research, the limited research in this area has shown promising results toward a theory of TRP metabolite and gut microbial cross talk in postnatal microbial development and immunity. In their murine model of transient gestational colonization (with a microbial strain that does not colonize the intestine), Gomez de Aguero et al. demonstrated that TRP, KYN, and IDO were present in breast milk of lactating dams who were germ-free but had been exposed to gestational colonization with an *Escherichia coli* strain (114). The combination of prenatal microbial exposure and postnatal nursing caused elevation in group 3 innate lymphoid cells (ILC3, counterparts to Th17 cells) and mononuclear cells of the neonatal immune system following vaginal birth. This TRP metabolite influence on neonatal immunity *via* breast milk following gestational microbial exposure is highly suggestive of maternal microbial programming of infant immune system development through the KYN-IDO pathway. New evidence is also emerging in human infants. In a cohort of initially breastfed infants, Hill et al. corroborated fecal microbial composition with the urine metabolome during the neonatal time period, finding a high correlation between the two systems (115). Metabolites from TRP metabolism detected in urine over the first 24 weeks of life significantly differentiated preterm and full-term neonates, and neonates born vaginally versus by cesarean section. Observed variation in TRP metabolism in newborns according to gestational age and birth mode also points to the influence of prenatal exposures and the birth process.

Once an infant is weaned off breast milk, solid food becomes a source for TRP. The Desbonnet et al. study, where antibiotics had been administered orally to pups once they were weaned off breast milk, reported reduced fecal microbial species richness and the following changes to serum levels: elevated TRP, reduced KYN, and higher KYNA/KYN and lower KYN/TRP ratios (92). These findings tell us that gut dysbiosis induced after breastfeeding also has the capacity to influence KYN metabolism. Of note, the reduced KYN/TRP ratio is in the same direction as that found in sera of children with persistent food allergy (83).

## Conclusion

Accumulating evidence is solidifying the role of the KYN-IDO pathway as an immunosuppressive pathway, which exhibits tolerogenic effects in response to stimuli through T-cell suppression, anergy, differentiation, and apoptosis. This immunosuppressive effect serves to suppress the overactive response of the immune system to various allergens. In the case of allergy, IDO is activated in response to allergen-induced immune activation, with the resultant production of KYN and KYN metabolites, and induction of tolerance. Most critically though, increasing evidence on germ-free mice and other early-life microbiome studies show that gut microbiota are key in determining the functioning of the KYN-IDO pathway with its broad range of activities.

Evidence of gut microbial influence on TRP concentrations and IDO activity raises the question: *could targeting the gut microbiome to modulate TRP metabolism and the KYN pathway treat or prevent diseases—specifically allergy?* Studies of *Bifidobacterium infantis* have reported increases of TRP concentrations and KYN metabolites after colonization (92, 116). In contrast, the addition of the *Lactobacillus johnsonii* probiotic has reduced IDO activity in clinical trials (117, 118). No doubt, the effectiveness of these probiotic interventions requires further testing, but one must appreciate the existence of critical windows in immune system development during infancy in relation to gut microbial colonization and stimulation of the KYN pathway. A better understanding of these early-life microbial processes is required, namely of how maternal microbiota and KYN metabolites shape the infant gut microbiome and how infant gut dysbiosis affects TRP metabolism. There is a clinical relevance to this research, with the capacity to intervene in the prevention of allergic and other immune-related diseases.

## Author Contributions

AL conducted the literature search and wrote the first draft. AK proposed the research question and provided funding. Both AK and YY edited the final version.

## Conflict of Interest Statement

The authors declare that the research was conducted in the absence of any commercial or financial relationships that could be construed as a potential conflict of interest.

## References

[1] Mellor AL Munn DH Tryptophan catabolism and regulation of adaptive immunity. J Immunol (2003) 170(12):5809–13. 10.4049/jimmunol.170.12.5809 12794104

[2] Thornton C Holt PG Development of allergy and atopy. In: Kay AB Kaplan AP Bousquet J Holt PG editors. Allergy and Allergic Diseases. (Vol. 1)2nd ed. Oxford, UK:Wiley-Blackwell (2008). p. 23–47.

[3] Bubnoff D Bieber T The indoleamine 2,3-dioxygenase (IDO) pathway controls allergy. Allergy (2012) 67(6):718–25. 10.1111/j.1398-9995.2012.02830.x 22519427

[4] Xu H Oriss TB Fei M Henry AC Melgert BN Chen L Indoleamine 2,3-dioxygenase in lung dendritic cells promotes Th2 responses and allergic inflammation. Proc Natl Acad Sci U S A (2008) 105(18):6690–5. 10.1073/pnas.0708809105 18436652 PMC2373348

[5] Slump P Schreuder HAW Determination of tryptophan in foods. Anal Biochem (1969) 27(1):182–6. 10.1016/0003-2697(69)90231-0 5407875

[6] Keszthelyi D Troost FJ Masclee AAM Understanding the role of tryptophan and serotonin metabolism in gastrointestinal function. Neurogastroenterol Motil (2009) 21(12):1239–49. 10.1111/j.1365-2982.2009.01370.x 19650771

[7] Kennedy PJ Cryan JF Dinan TG Clarke G. Kynurenine pathway metabolism and the microbiota-gut-brain axis. Neuropharmacology (2017) 112:399–412. 10.1016/j.neuropharm.2016.07.002 27392632

[8] Heitger A Regulation of expression and function of IDO in human dendritic cells. Curr Med Chem (2011) 18(15):2222–33. 10.2174/092986711795656018 21517757

[9] Blaschitz A Gauster M Fuchs D Lang I Maschke P Ulrich D Vascular endothelial expression of indoleamine 2,3-dioxygenase 1 forms a positive gradient towards the feto-maternal interface. PLoS One (2011) 6(7):e21774. 10.1371/journal.pone.0021774 21755000 PMC3130744

[10] Fernstrom JD. Branched-chain amino acids and brain function. J Nutr (2005) 135(6 Suppl):1539S–46S. 15930466 10.1093/jn/135.6.1539S

[11] Nakamura K Hasegawa H. Production and peripheral roles of 5-HTP, a precursor of serotonin. Int J Tryptophan Res (2009) 2:37–43. 22084581 10.4137/ijtr.s1022 PMC3195225

[12] Bornstein JC Serotonin in the gut: what does it do? Front Neurosci (2012) 6:16 10.3389/fnins.2012.00016 22347162 PMC3272651

[13] Chen Y Guillemin GJ. Kynurenine pathway metabolites in humans: disease and healthy states. Int J Tryptophan Res (2009) 2:1–19. 22084578 10.4137/ijtr.s2097 PMC3195227

[14] Yang Y Sauve AA. NAD+ metabolism: bioenergetics, signaling and manipulation for therapy. Biochim Biophys Acta (2016) 1864(12):1787–800. 10.1016/j.bbapap.2016.06.014 27374990 PMC5521000

[15] Li JS Han Q Fang J Rizzi M James AA Li J. Biochemical mechanisms leading to tryptophan 2,3-dioxygenase activation. Arch Insect Biochem Physiol (2007) 64(2):74–87. 10.1002/arch.20159 17212352 PMC2565576

[16] Pantouris G Serys M Yuasa HJ Ball HJ Mowat CG. Human indoleamine 2,3-dioxygenase-2 has substrate specificity and inhibition characteristics distinct from those of indoleamine 2,3-dioxygenase-1. Amino Acids (2014) 46(9):2155–63. 10.1007/s00726-014-1766-3 24875753

[17] Lob S Konigsrainer A Rammensee H-G Opelz G Terness P. Inhibitors of indoleamine-2,3-dioxygenase for cancer therapy: can we see the wood for the trees? Nat Rev Cancer (2009) 9(6):445–52. 10.1038/nrc2639 19461669

[18] Witkiewicz A Williams TK Cozzitorto J Durkan B Showalter SL Yeo CJ Expression of indoleamine 2,3-dioxygenase in metastatic pancreatic ductal adenocarcinoma recruits regulatory T cells to avoid immune detection. J Am Coll Surg (2008) 206(5):849–54; discussion 854–6. 10.1016/j.jamcollsurg.2007.12.014 18471709

[19] Metz R DuHadaway JB Kamasani U Laury-Kleintop L Muller AJ Prendergast GC. Novel tryptophan catabolic enzyme IDO2 is the preferred biochemical target of the antitumor indoleamine 2,3-dioxygenase inhibitory compound d-1-methyl-tryptophan. Cancer Res (2007) 67(15):7082–7. 10.1158/0008-5472.CAN-07-1872 17671174

[20] Sun T Chen X-H Tang Z-D Cai J Wang X-Y Wang S-C Novel 1-alkyl-tryptophan derivatives downregulate IDO1 and IDO2 mRNA expression induced by interferon-gamma in dendritic cells. Mol Cell Biochem (2010) 342(1–2):29–34. 10.1007/s11010-010-0465-y 20424892

[21] Croitoru-Lamoury J Lamoury FMJ Caristo M Suzuki K Walker D Takikawa O Interferon-γ regulates the proliferation and differentiation of mesenchymal stem cells via activation of indoleamine 2,3 dioxygenase (IDO). PLoS One (2011) 6(2):e14698. 10.1371/journal.pone.0014698 21359206 PMC3040184

[22] Fujigaki S Saito K Takemura M Fujii H Wada H Noma A Species differences in l-tryptophan-kynurenine pathway metabolism: quantification of anthranilic acid and its related enzymes. Arch Biochem Biophys (1998) 358(2):329–35. 10.1006/abbi.1998.0861 9784247

[23] Takikawa O Yoshida R Kido R Hayaishi O. Tryptophan degradation in mice initiated by indoleamine 2,3-dioxygenase. J Biol Chem (1986) 261(8):3648–53. 2419335

[24] Yoshida R Imanishi J Oku T Kishida T Hayaishi O. Induction of pulmonary indoleamine 2,3-dioxygenase by interferon. Proc Natl Acad Sci U S A (1981) 78(1):129–32. 10.1073/pnas.78.1.129 6165986 PMC319004

[25] Ball HJ Yuasa HJ Austin CJD Weiser S Hunt NH. Indoleamine 2,3-dioxygenase-2; a new enzyme in the kynurenine pathway. Int J Biochem Cell Biol (2009) 41(3):467–71. 10.1016/j.biocel.2008.01.005 18282734

[26] Metz R Rust S DuHadaway JB Mautino MR Munn DH Vahanian NN IDO inhibits a tryptophan sufficiency signal that stimulates mTOR: a novel IDO effector pathway targeted by d-1-methyl-tryptophan. Oncoimmunology (2014) 1(9):1460–8. 10.4161/onci.21716 PMC3525601 23264892

[27] Merlo LMF Pigott E DuHadaway JB Grabler S Metz R Prendergast GC IDO2 is a critical mediator of autoantibody production and inflammatory pathogenesis in a mouse model of autoimmune arthritis. J Immunol (2014) 192(5):2082–90. 10.4049/jimmunol.1303012 24489090 PMC3947779

[28] Lob S Konigsrainer A Schafer R Rammensee H-G Opelz G Terness P. Levo- but not dextro-1-methyl tryptophan abrogates the IDO activity of human dendritic cells. Blood (2008) 111(4):2152–4. 10.1182/blood-2007-10-116111 18045970

[29] Merlo LMF Grabler S DuHadaway JB Pigott E Manley K Prendergast GC Therapeutic antibody targeting of indoleamine-2,3-dioxygenase (IDO2) inhibits autoimmune arthritis. Clin Immunol (2017) 179:8–16. 10.1016/j.clim.2017.01.016 28223071 PMC5466478

[30] Adams S Braidy N Bessede A Brew BJ Grant R Teo C The kynurenine pathway in brain tumor pathogenesis. Cancer Res (2012) 72(22):5649–57. 10.1158/0008-5472.CAN-12-0549 23144293

[31] Xu H Zhang G-X Ciric B Rostami A. IDO: a double-edged sword for T(H)1/T(H)2 regulation. Immunol Lett (2008) 121(1):1–6. 10.1016/j.imlet.2008.08.008 18824197 PMC2628165

[32] Yeung AWS Terentis AC King NJC Thomas SR. Role of indoleamine 2,3-dioxygenase in health and disease. Clin Sci (Lond) (2015) 129(7):601–72. 10.1042/CS20140392 26186743

[33] Christmas DM Potokar JP Davies SJ A biological pathway linking inflammation and depression: activation of indoleamine 2,3-dioxygenase. Neuropsychiatr Dis Treat (2011) 7:431–9. 10.2147/NDT.S17573 21792309 PMC3140295

[34] Nakamura T Shinno H Ischiara A. Insulin and glucagon as a new regulator system for tryptophan oxygenase activity demonstrated in primary cultured rat hepatocytes. J Biol Chem (1980) 255(16):7533–5. 6249804

[35] Schmidt SK Muller A Heseler K Woite C Spekker K MacKenzie CR Antimicrobial and immunoregulatory properties of human tryptophan 2,3-dioxygenase. Eur J Immunol (2009) 39(10):2755–64. 10.1002/eji.200939535 19637229

[36] Larkin PB Sathyasaikumar KV Notarangelo FM Funakoshi H Nakamura T Schwarcz R Tryptophan 2,3-dioxygenase and indoleamine 2,3-dioxygenase 1 make separate, tissue-specific contributions to basal and inflammation-induced kynurenine pathway metabolism in mice. Biochim Biophys Acta (2016) 1860(11 Pt A):2345–54. 10.1016/j.bbagen.2016.07.002 27392942 PMC5808460

[37] Mellor AL Munn DH. IDO expression by dendritic cells: tolerance and tryptophan catabolism. Nat Rev Immunol (2004) 4(10):762–74. 10.1038/nri1457 15459668

[38] Wallet MA Sen P Tisch R Immunoregulation of dendritic cells. Clin Med Res (2005) 3(3):166–75. 10.3121/cmr.3.3.166 16160071 PMC1237158

[39] Pfefferkorn ER Interferon gamma blocks the growth of *Toxoplasma gondii* in human fibroblasts by inducing the host cells to degrade tryptophan. Proc Natl Acad Sci U S A (1984) 81(3):908–12. 10.1073/pnas.81.3.908 6422465 PMC344948

[40] Pfefferkorn ER Guyre PM. Inhibition of growth of *Toxoplasma gondii* in cultured fibroblasts by human recombinant gamma interferon. Infect Immun (1984) 44(2):211–6. 6425215 10.1128/iai.44.2.211-216.1984 PMC263502

[41] Badawy AAB Namboodiri AMA Moffett JR. The end of the road for the tryptophan depletion concept in pregnancy and infection. Clin Sci (2016) 130(15):1327–33. 10.1042/CS20160153 27358028 PMC4926258

[42] Cervenka I Agudelo LZ Ruas JL Kynurenines: tryptophan’s metabolites in exercise, inflammation, and mental health. Science (2017) 357(6349):eaaf9794 10.1126/science.aaf9794 28751584

[43] Munn DH. Prevention of allogeneic fetal rejection by tryptophan catabolism. Science (1998) 281(5380):1191–3. 9712583 10.1126/science.281.5380.1191

[44] Munn DH Sharma MD Lee JR Jhaver KG Johnson TS Keskin DB Potential regulatory function of human dendritic cells expressing indoleamine 2,3-dioxygenase. Science (2002) 297(5588):1867–70. 10.1126/science.1073514 12228717

[45] Munn DH Shafizadeh E Attwood JT Bondarev I Pashine A Mellor AL. Inhibition of T cell proliferation by macrophage tryptophan catabolism. J Exp Med (1999) 189(9):1363–72. 10.1084/jem.189.9.1363 10224276 PMC2193062

[46] Munn DH Sharma MD Mellor AL Ligation of B7-1/B7-2 by human CD4+ T cells triggers indoleamine 2,3-dioxygenase activity in dendritic cells. J Immunol (2004) 172(7):4100–10. 10.4049/jimmunol.172.7.4100 15034022

[47] Mellor AL Chandler P Baban B Hansen AM Marshall B Pihkala J Specific subsets of murine dendritic cells acquire potent T cell regulatory functions following CTLA4-mediated induction of indoleamine 2,3 dioxygenase. Int Immunol (2004) 16(10):1391–401. 10.1093/intimm/dxh140 15351783

[48] Lee GK Park HJ Macleod M Chandler P Munn DH Mellor AL. Tryptophan deprivation sensitizes activated T cells to apoptosis prior to cell division. Immunology (2002) 107(4):452–60. 10.1046/j.1365-2567.2002.01526.x 12460190 PMC1782830

[49] Munn DH Sharma MD Baban B Harding HP Zhang Y Ron D GCN2 kinase in T cells mediates proliferative arrest and anergy induction in response to indoleamine 2,3-dioxygenase. Immunity (2005) 22(5):633–42. 10.1016/j.immuni.2005.03.013 15894280

[50] Hinnebusch AG. The eIF-2 alpha kinases: regulators of protein synthesis in starvation and stress. Semin Cell Biol (1994) 5(6):417–26. 10.1006/scel.1994.1049 7711290

[51] Rao RV Ellerby HM Bredesen DE. Coupling endoplasmic reticulum stress to the cell death program. Cell Death Differ (2004) 11(4):372–80. 10.1038/sj.cdd.4401378 14765132

[52] Fallarino F Grohmann U Vacca C Bianchi R Orabona C Spreca A T cell apoptosis by tryptophan catabolism. Cell Death Differ (2002) 9(10):1069–77. 10.1038/sj.cdd.4401073 12232795

[53] Pallotta MT Orabona C Volpi C Vacca C Belladonna ML Bianchi R Indoleamine 2,3-dioxygenase is a signaling protein in long-term tolerance by dendritic cells. Nat Immunol (2011) 12(9):870–8. 10.1038/ni.2077 21804557

[54] Frumento G Rotondo R Tonetti M Damonte G Benatti U Ferrara GB. Tryptophan-derived catabolites are responsible for inhibition of T and natural killer cell proliferation induced by indoleamine 2,3-dioxygenase. J Exp Med (2002) 196(4):459–68. 10.1084/jem.20020121 12186838 PMC2196046

[55] Terness P Bauer TM Rose L Dufter C Watzlik A Simon H Inhibition of allogeneic T cell proliferation by indoleamine 2,3-dioxygenase-expressing dendritic cells: mediation of suppression by tryptophan metabolites. J Exp Med (2002) 196(4):447–57. 10.1084/jem.20020052 12186837 PMC2196057

[56] Lee S-M Lee Y-S Choi J-H Park S-G Choi I-W Joo Y-D Tryptophan metabolite 3-hydroxyanthranilic acid selectively induces activated T cell death via intracellular GSH depletion. Immunol Lett (2010) 132(1–2):53–60. 10.1016/j.imlet.2010.05.008 20570696

[57] Fallarino F Grohmann U You S McGrath BC Cavener DR Vacca C The combined effects of tryptophan starvation and tryptophan catabolites down-regulate T cell receptor zeta-chain and induce a regulatory phenotype in naive T cells. J Immunol (2006) 176(11):6752–61. 10.4049/jimmunol.176.11.6752 16709834

[58] Boasso A Herbeuval J-P Hardy AW Winkler C Shearer GM. Regulation of indoleamine 2,3-dioxygenase and tryptophanyl-tRNA-synthetase by CTLA-4-Fc in human CD4+ T cells. Blood (2005) 105(4):1574–81. 10.1182/blood-2004-06-2089 15466932

[59] Mezrich JD Fechner JH Zhang X Johnson BP Burlingham WJ Bradfield CA. An interaction between kynurenine and the aryl hydrocarbon receptor can generate regulatory T cells. J Immunol (2010) 185(6):3190–8. 10.4049/jimmunol.0903670 20720200 PMC2952546

[60] Nguyen NT Kimura A Nakahama T Chinen I Masuda K Nohara K Aryl hydrocarbon receptor negatively regulates dendritic cell immunogenicity via a kynurenine-dependent mechanism. Proc Natl Acad Sci U S A (2010) 107(46):19961–6. 10.1073/pnas.1014465107 21041655 PMC2993339

[61] Vogel CFA Goth SR Dong B Pessah IN Matsumura F. Aryl hydrocarbon receptor signaling mediates expression of indoleamine 2,3-dioxygenase. Biochem Biophys Res Commun (2008) 375(3):331–5. 10.1016/j.bbrc.2008.07.156 18694728 PMC2583959

[62] DiNatale BC Murray IA Schroeder JC Flaveny CA Lahoti TS Laurenzana EM Kynurenic acid is a potent endogenous aryl hydrocarbon receptor ligand that synergistically induces interleukin-6 in the presence of inflammatory signaling. Toxicol Sci (2010) 115(1):89–97. 10.1093/toxsci/kfq024 20106948 PMC2855350

[63] Grohmann U Orabona C Fallarino F Vacca C Calcinaro F Falorni A CTLA-4-Ig regulates tryptophan catabolism in vivo. Nat Immunol (2002) 3(11):1097–101. 10.1038/ni846 12368911

[64] Fallarino F Grohmann U Hwang KW Orabona C Vacca C Bianchi R Modulation of tryptophan catabolism by regulatory T cells. Nat Immunol (2003) 4(12):1206–12. 10.1038/ni1003 14578884

[65] Odemuyiwa SO Ghahary A Li Y Puttagunta L Lee JE Musat-Marcu S Cutting edge: human eosinophils regulate T cell subset selection through indoleamine 2,3-dioxygenase. J Immunol (2004) 173(10):5909–13. 10.4049/jimmunol.173.10.5909 15528322

[66] Molano A Illarionov PA Besra GS Putterman C Porcelli SA. Modulation of invariant natural killer T cell cytokine responses by indoleamine 2,3-dioxygenase. Immunol Lett (2008) 117(1):81–90. 10.1016/j.imlet.2007.12.013 18272236 PMC2367367

[67] Hayashi T Beck L Rossetto C Gong X Takikawa O Takabayashi K Inhibition of experimental asthma by indoleamine 2,3-dioxygenase. J Clin Invest (2004) 114(2):270–9. 10.1172/JCI21275 15254594 PMC449749

[68] Zelante T Iannitti RG Cunha C De Luca A Giovannini G Pieraccini G Tryptophan catabolites from microbiota engage aryl hydrocarbon receptor and balance mucosal reactivity via interleukin-22. Immunity (2013) 39(2):372–85. 10.1016/j.immuni.2013.08.003 23973224

[69] Halim TYF Steer CA Mathä L Gold MJ Martinez-Gonzalez I McNagny KM Group 2 innate lymphoid cells are critical for the initiation of adaptive T helper 2 cell-mediated allergic lung inflammation. Immunity (2014) 40(3):425–35. 10.1016/j.immuni.2014.01.011 24613091 PMC4210641

[70] Barlow JL Bellosi A Hardman CS Drynan LF Wong SH Cruickshank JP Innate IL-13-producing nuocytes arise during allergic lung inflammation and contribute to airways hyperreactivity. J Allergy ClinImmunol (2012) 129(1): 191–8.e1–4. 10.1016/j.jaci.2011.09.041 22079492

[71] Chang Y-J Kim HY Albacker LA Baumgarth N McKenzie ANJ Smith DE Innate lymphoid cells mediate influenza-induced airway hyper-reactivity independently of adaptive immunity. Nat Immunol (2011) 12(7):631–8. 10.1038/ni.2045 21623379 PMC3417123

[72] Hams E Locksley RM McKenzie ANJ Fallon PG. Cutting edge: IL-25 elicits innate lymphoid type 2 and type II NKT cells that regulate obesity in mice. J Immunol (2013) 191(11):5349–53. 10.4049/jimmunol.1301176 24166975 PMC3847854

[73] Halim TYF Krauss RH Sun AC Takei F. Lung natural helper cells are a critical source of Th2 cell-type cytokines in protease allergen-induced airway inflammation. Immunity (2012) 36(3):451–63. 10.1016/j.immuni.2011.12.020 22425247

[74] MacKenzie CR Heseler K Muller A Daubener W. Role of indoleamine 2,3-dioxygenase in antimicrobial defence and immuno-regulation: tryptophan depletion versus production of toxic kynurenines. Curr Drug Metab (2007) 8(3):237–44. 10.2174/138920007780362518 17430112

[75] Adams O Besken K Oberdorfer C MacKenzie CR Takikawa O Daubener W. Role of indoleamine-2,3-dioxygenase in alpha/beta and gamma interferon-mediated antiviral effects against herpes simplex virus infections. J Virol (2004) 78(5):2632–6. 10.1128/JVI.78.5.2632-2636.2004 14963171 PMC369218

[76] Taylor MW Feng GS. Relationship between interferon-gamma, indoleamine 2,3-dioxygenase, and tryptophan catabolism. FASEB J (1991) 5(11):2516–22. 1907934

[77] Puccetti P Grohmann U. IDO and regulatory T cells: a role for reverse signalling and non-canonical NF-kappaB activation. Nat Rev Immunol (2007) 7(10):817–23. 10.1038/nri2163 17767193

[78] Suzue K Asai T Takeuchi T Koyasu S. In vivo role of IFN-gamma produced by antigen-presenting cells in early host defense against intracellular pathogens. Eur J Immunol (2003) 33(10):2666–75. 10.1002/eji.200323292 14515250

[79] Fricke I Mitchell D Mittelstadt J Lehan N Heine H Goldmann T Mycobacteria induce IFN-gamma production in human dendritic cells via triggering of TLR2. J Immunol (2006) 176(9):5173–82. 10.4049/jimmunol.176.9.5173 16621981

[80] Bubnoff Von D Matz H Frahnert C Rao ML Hanau D la Salle de H FcepsilonRI induces the tryptophan degradation pathway involved in regulating T cell responses. J Immunol (2002) 169(4):1810–6. 10.4049/jimmunol.169.4.1810 12165503

[81] Bubnoff von D la Salle de H Wessendorf J Koch S Hanau D Bieber T. Antigen-presenting cells and tolerance induction. Allergy (2002) 57(1):2–8. 10.1046/j.0105-4538.2001.00001.x-i4 11991283

[82] Bubnoff von D Fimmers R Bogdanow M Matz H Koch S Bieber T. Asymptomatic atopy is associated with increased indoleamine 2,3-dioxygenase activity and interleukin-10 production during seasonal allergen exposure. Clin Exp Allergy (2004) 34(7):1056–63. 10.1111/j.1365-2222.2004.01984.x 15248850

[83] Buyuktiryaki B Sahiner UM Girgin G Birben E Soyer OU Cavkaytar O Low indoleamine 2,3-dioxygenase activity in persistent food allergy in children. Allergy (2016) 71(2):258–66. 10.1111/all.12785 26449488

[84] Bubnoff von D Bezold G Matz H Hanau D la Salle de H Bieber T. Quantification of indoleamine 2,3-dioxygenase gene induction in atopic and non-atopic monocytes after ligation of the high-affinity receptor for IgE, Fc(epsilon)RI and interferon-gamma stimulation. Clin Exp Immunol (2003) 132(2):247–53. 10.1046/j.1365-2249.2003.02125.x 12699412 PMC1808689

[85] Taher YA Piavaux BJA Gras R van Esch BCAM Hofman GA Bloksma N Indoleamine 2,3-dioxygenase–dependent tryptophan metabolites contribute to tolerance induction during allergen immunotherapy in a mouse model. J Allergy ClinImmunol (2008) 121(4):983–91.e2. 10.1016/j.jaci.2007.11.021 18179817

[86] Bussmann C Xia J Allam JP Maintz L Bieber T Novak N. Early markers for protective mechanisms during rush venom immunotherapy. Allergy (2010) 65(12):1558–65. 10.1111/j.1398-9995.2010.02430.x 20584008

[87] Moingeon P Batard T Fadel R Frati F Sieber J Van Overtvelt L. Immune mechanisms of allergen-specific sublingual immunotherapy. Allergy (2006) 61(2):151–65. 10.1111/j.1398-9995.2006.01002.x 16409190

[88] Favre D Mold J Hunt PW Kanwar B Loke P Seu L Tryptophan catabolism by indoleamine 2,3-dioxygenase 1 alters the balance of TH17 to regulatory T cells in HIV disease. Sci Transl Med (2010) 2(32):32ra36. 10.1126/scitranslmed.3000632 20484731 PMC3034445

[89] Fuertig R Azzinnari D Bergamini G Cathomas F Sigrist H Seifritz E Mouse chronic social stress increases blood and brain kynurenine pathway activity and fear behaviour: both effects are reversed by inhibition of indoleamine 2,3-dioxygenase. Brain Behav Immun (2016) 54:59–72. 10.1016/j.bbi.2015.12.020 26724575

[90] Arreola R Becerril-Villanueva E Cruz-Fuentes C Velasco-Velazquez MA Garces-Alvarez ME Hurtado-Alvarado G Immunomodulatory effects mediated by serotonin. J Immunol Res (2015) 2015:354957. 10.1155/2015/354957 25961058 PMC4417587

[91] Agudelo LZ Femenia T Orhan F Porsmyr-Palmertz M Goiny M Martinez-Redondo V Skeletal muscle PGC-1alpha1 modulates kynurenine metabolism and mediates resilience to stress-induced depression. Cell (2014) 159(1):33–45. 10.1016/j.cell.2014.07.051 25259918

[92] Desbonnet L Clarke G Traplin A O’Sullivan O Crispie F Moloney RD Gut microbiota depletion from early adolescence in mice: implications for brain and behaviour. Brain Behav Immun (2015) 48:165–73. 10.1016/j.bbi.2015.04.004 25866195

[93] Kennedy PJ Irritable bowel syndrome: a microbiome-gut-brain axis disorder? WJG (2014) 20(39):14105–25. 10.3748/wjg.v20.i39.14105 25339800 PMC4202342

[94] Backhed F Ley RE Sonnenburg JL Peterson DA Gordon JI. Host-bacterial mutualism in the human intestine. Science (2005) 307(5717):1915–20. 10.1126/science.1104816 15790844

[95] Hooper LV Wong MH Thelin A Hansson L Falk PG Gordon JI. Molecular analysis of commensal host-microbial relationships in the intestine. Science (2001) 291(5505):881–4. 10.1126/science.291.5505.881 11157169

[96] Wikoff WR Anfora AT Liu J Schultz PG Lesley SA Peters EC Metabolomics analysis reveals large effects of gut microflora on mammalian blood metabolites. Proc Natl Acad Sci U S A (2009) 106(10):3698–703. 10.1073/pnas.0812874106 19234110 PMC2656143

[97] Aidy El S Dinan TG Cryan JF Immune modulation of the brain-gut-microbe axis. Front Microbiol (2014) 5:146 10.3389/fmicb.2014.00146 24778631 PMC3985034

[98] Shoaie S Ghaffari P Kovatcheva-Datchary P Mardinoglu A Sen P Pujos-Guillot E Quantifying diet-induced metabolic changes of the human gut microbiome. Cell Metab (2015) 22(2):320–31. 10.1016/j.cmet.2015.07.001 26244934

[99] Clarke SF Murphy EF O’Sullivan O Ross RP O’Toole PW Shanahan F Targeting the microbiota to address diet-induced obesity: a time dependent challenge. PLoS One (2013) 8(6):e65790. 10.1371/journal.pone.0065790 23762426 PMC3676335

[100] Bercik P Verdu EF Foster JA Macri J Potter M Huang X Chronic gastrointestinal inflammation induces anxiety-like behavior and alters central nervous system biochemistry in mice. Gastroenterology (2010) 139(6): 2102–12.e1. 10.1053/j.gastro.2010.06.063 20600016

[101] Reigstad CS Salmonson CE Rainey JF Szurszewski JH Linden DR Sonnenburg JL Gut microbes promote colonic serotonin production through an effect of short-chain fatty acids on enterochromaffin cells. FASEB J (2015) 29(4):1395–403. 10.1096/fj.14-259598 25550456 PMC4396604

[102] Yano JM Yu K Donaldson GP Shastri GG Ann P Ma L Indigenous bacteria from the gut microbiota regulate host serotonin biosynthesis. Cell (2015) 161(2):264–76. 10.1016/j.cell.2015.02.047 25860609 PMC4393509

[103] Bessede A Gargaro M Pallotta MT Matino D Servillo G Brunacci C Aryl hydrocarbon receptor control of a disease tolerance defence pathway. Nature (2014) 511(7508):184–90. 10.1038/nature13323 24930766 PMC4098076

[104] Jaronen M Quintana FJ. Immunological relevance of the coevolution of IDO1 and AHR. Front Immunol (2014) 5:521. 10.3389/fimmu.2014.00521 25368620 PMC4202789

[105] García-Lara J Weihs F Ma X Walker L Chaudhuri RR Kasturiarachchi J Supramolecular structure in the membrane of *Staphylococcus aureus*. Proc Natl Acad Sci U S A (2015) 112(51):15725–30. 10.1073/pnas.1509557112 26644587 PMC4697411

[106] Kawai T Akira S. The role of pattern-recognition receptors in innate immunity: update on Toll-like receptors. Nat Immunol (2010) 11(5):373–84. 10.1038/ni.1863 20404851

[107] Erejuwa O Sulaiman S Wahab M. Modulation of gut microbiota in the management of metabolic disorders: the prospects and challenges. Int J Mol Sci (2014) 15(3):4158–88. 10.3390/ijms15034158 24608927 PMC3975390

[108] Martin R Makino H Cetinyurek Yavuz A Ben-Amor K Roelofs M Ishikawa E Early-life events, including mode of delivery and type of feeding, siblings and gender, shape the developing gut microbiota. PLoS One (2016) 11(6):e0158498. 10.1371/journal.pone.0158498 27362264 PMC4928817

[109] Nuriel-Ohayon M Neuman H Koren O Microbial changes during pregnancy, birth, and infancy. Front Microbiol (2016) 7(104):237ra65 10.3389/fmicb.2016.01031 27471494 PMC4943946

[110] Koleva PT Bridgman SL Kozyrskyj AL. The infant gut microbiome: evidence for obesity risk and dietary intervention. Nutrients (2015) 7(4):2237–60. 10.3390/nu7042237 25835047 PMC4425142

[111] Bridgman SL Kozyrskyj AL Scott JA Becker AB Azad MB Gut microbiota and allergic disease in children. Ann Allergy Asthma Immunol (2016) 116(2):99–105. 10.1016/j.anai.2015.10.001 26815703

[112] Round JL Mazmanian SK. The gut microbiota shapes intestinal immune responses during health and disease. Nat Rev Immunol (2009) 9(5):313–23. 10.1038/nri2515 19343057 PMC4095778

[113] Strasser B Geiger D Schauer M Gostner JM Gatterer H Burtscher M Probiotic supplements beneficially affect tryptophan–kynurenine metabolism and reduce the incidence of upper respiratory tract infections in trained athletes: a randomized, double-blinded, placebo-controlled trial. Nutrients (2016) 8(11):752 10.3390/nu8110752 PMC5133134 27886064

[114] Gomez de Aguero M Ganal-Vonarburg SC Fuhrer T Rupp S Uchimura Y Li H The maternal microbiota drives early postnatal innate immune development. Science (2016) 351(6279):1296–302. 10.1126/science.aad2571 26989247

[115] Hill CJ Lynch DB Murphy K Ulaszewska M Jeffery IB O’Shea CA Evolution of gut microbiota composition from birth to 24 weeks in the INFANTMET Cohort. Microbiome (2017) 5(1):4. 10.1186/s40168-016-0213-y 28095889 PMC5240274

[116] Desbonnet L Garrett L Clarke G Bienenstock J Dinan TG. The probiotic *Bifidobacteria infantis*: an assessment of potential antidepressant properties in the rat. J Psychiatr Res (2008) 43(2):164–74. 10.1016/j.jpsychires.2008.03.009 18456279

[117] Valladares RB Graves C Wright K Gardner CL Lorca GL Gonzalez CF H(2)O(2) production rate in *Lactobacillus johnsonii* is modulated via the interplay of a heterodimeric flavin oxidoreductase with a soluble 28 Kd PAS domain containing protein. Front Microbiol (2015) 6:716 10.3389/fmicb.2015.00716 26236298 PMC4500961

[118] Marcial GE Ford AL Haller MJ Gezan SA Harrison NA Cai D *Lactobacillus johnsonii* N6.2 modulates the host immune responses: a double-blind, randomized trial in healthy adults. Front Immunol (2017) 8:655. 10.3389/fimmu.2017.00655 28659913 PMC5466969

